# Supplementary material for: Subdomains of Post-COVID Syndrome (PCS) – a population-based study
Source: BMC Infect Dis. 2025 Aug 26;25:1072. doi: 10.1186/s12879-025-11368-6 (PMC12382122; doi:10.1186/s12879-025-11368-6)
Supplement: Supplementary file 1 — Supplementary Material 1. [file 12879_2025_11368_MOESM1_ESM.docx]

# **Subdomains of Post-COVID Syndrome (PCS) – A Population-Based Study (Sabrina Ballhausen-Lübcker, Anne-Kathrin Ruß, et al.)**

# **SUPPLEMENTARY MATERIAL**

## **COVIDOM data acquisition**

Before visiting a study center, COVIDOM participants were asked to answer and return a questionnaire, either paper-based or online. In addition to basic personal, demographic and medical characteristics, the questionnaire included questions about the circumstances of the acute SARS‑CoV-2 infection, the subsequent course of disease, and the lifestyle and current health status of the patient, particularly the persistence of COVID-19 symptoms.

During the visit to the study center, patients were examined clinically and an additional anamnesis was taken, drawing upon various validated questionnaires such as PHQ-8 (depression), GAD-7 (anxiety disorder), FACIT-F and CFS (fatigue), BRS (resilience), MoCA (cognitive function), PSS (stress), mMRC and MDP (dyspnea) as well as EQ-5D-5L (quality of life). Diagnostic measures included the assessment of body height and weight, bioelectric impedance scanning, and the control of patient vitality parameters. More specific tests were carried out from different medical disciplines, including ear-nose-throat (chemosensory and endoscopy), neurology (neurological examinations), pneumology (standardized spirometry, including body plethysmography, and diffusion capacity), cardiology (indirect blood pressure measurement, ECG, and echocardiography), hepatology (hepatic sonography and elastography), and geriatrics (6-minute walking test for probands >65 years of age).

After a final discussion with their doctor, every patient received a medical report containing their test results and, if necessary, individual health-related recommendations. All data collected at the COVIDOM study centers in Berlin, Kiel and Würzburg were deposited in a central database.

## **Statistical analysis**

CART analysis

Classification and Regression Tree (CART) analysis as implemented in R package *rpart* was used to identify subsets of the 12 PCS score-constituting symptom complexes that were potentially more specific to individual resilience and acute phase COVID-19 severity as the main predictors of overall PCS severity. While either individual resilience or acute COVID-19 severity served as the respective target variable in one of two separate CART analyses, the binary symptom complex indicators were used as potential classifiers in both instances. Node purity was measured by the Gini index, and the number of cross-validations was set to 1,000. To avoid overfitting, the resulting trees were pruned until each terminal node comprised at least 5% of the samples.

Candidate symptom complexes for the subsequent construction of two novel, predictor-specific PCS scores were selected by the elbow method, drawing upon the importance values of individual symptom complexes as provided by *rpart*. The importance value of a CART classifier measures its discriminatory relevance among all splits in a tree. The elbow method, on the other hand, is a heuristic to determine the number and identity of relevant classifiers. It consists in plotting the importance values of the classifiers of interest by size and picking the elbow of the curve to demarcate the classifiers to use. In the present CART analysis, this led to importance value thresholds (i.e. elbows) of 14.3 and 22.5 for resilience and acute severity, respectively.

Construction of predictor-specific PCS scores

Based upon the results of the CART analysis, two novel PCS scores were constructed in sub-cohort Kiel-I (n=667) following the same procedure as pursued in the definition of the original PCS score (Bahmer et al. 2022). In brief, each CART-selected subset of symptom complexes was subjected to an iterative combination of k-means clustering and ordinal logistic regression analysis, treating the cluster affiliation of a participant as the respective outcome variable. Cluster number k was increased until the logistic regression models became sufficiently stable according to the Pearson correlation coefficient between the scores resulting from subsequent regression models. The regression coefficients of the final models served as weights in the subsequent PCS score definitions. Thresholds for the assignment of participants to clinically meaningful subgroups were derived for both scores by receiver operator curve (ROC) analysis, as described by Bahmer et al. (2022).

#### Post-hoc identification of predictors of novel PCS scores

Potential predictors of the two novel PCS scores were evaluated *post-hoc* by way of multiple ordinal logistic regression analysis with backward selection (threshold p<0.05) as described by Bahmer et al. (2022), treating the respective PCS score class (for definition, see Results section of the main text) as the outcome variable. Potential predictors were chosen from the 10 acute phase and general characteristics of COVIDOM participants that had been identified before as being significantly associated with the original PCS score (Bahmer et al. 2022). Missing values of predictor variables were imputed by multiple imputation assuming that missingness was completely at random. Two independent analyses were carried out in substantially expanded COVIDOM sub-cohorts Würzburg/Berlin (n=959) and Kiel-II (n=1,746), neither of which overlapped with sub-cohort Kiel-I in which the scores were constructed.

Reference

Bahmer T, Borzikowsky C, Lieb W, Horn A, Krist L, Fricke J, et al. Severity, predictors and clinical correlates of Post-COVID syndrome (PCS) in Germany: A prospective, multi-centre, population-based cohort study. eClinicalMedicine 2022;51:101549.

**Supplementary Table 1: Significant predictors of resilience-specific PCS-R score class in sub-cohorts Würzburg/Berlin and Kiel-II**

| **Predictor variable** | **Level** | **Regression coefficient** | | | **Odds ratio** | | **P value^d^** | |
| --- | --- | --- | --- | --- | --- | --- | --- | --- |
|  |  | **Estimate** | **Standard error** | **95% confidence interval** | **Estimate** | **95% confidence interval** | **Unadjusted** | **Adjusted** |
| *Würzburg/Berlin (n=884)** | | | | | | | | |
| Resilience (BRS) | scale | -0.460 | 0.126 | [-0.707; -0.213] | 0.631 | [0.493; 0.808] | 0.00032 | 0.0038 |
| No. serious or life-threatening symptoms^a^ | 1-3 | 0.203 | 0.252 | [-0.291; 0.697] | 1.225 | [0.748; 2.007] | 0.42 | n.a. |
|  | 4-6 | 0.548 | 0.318 | [-0.076; 1.171] | 1.729 | [0.927; 3.226] | 0.091 | n.a. |
|  | >7 | 0.991 | 0.335 | [0.333; 1.648] | 2.693 | [1.395; 5.198] | 0.0036 | 0.043 |
| Pre-existing neurologic or psychiatric disease | yes | 1.025 | 0.234 | [0.566; 1.483] | 2.786 | [1.761; 4.407] | <0.0001 | <0.0001 |
| General anxiousness | yes | 0.517 | 0.17 | [0.184; 0.850] | 1.677 | [1.203; 2.340] | 0.0024 | 0.029 |
| Pre-existing cardiovascular disease | yes | 0.697 | 0.228 | [0.251; 1.143] | 2.008 | [1.285; 3.136] | 0.0028 | 0.034 |
| No. symptoms^b^ | 3-5 | -1.034 | 0.479 | [-1.974; -0.095] | 0.355 | [0.139; 0.909] | 0.032 | 0.38 |
|  | 6-8 | 0.408 | 0.371 | [-0.319; 1.135] | 1.504 | [0.727; 3.113] | 0.27 | n.a. |
|  | >9 | 0.928 | 0.356 | [0.231; 1.625] | 2.531 | [1.260; 5.08] | 0.0092 | 0.11 |
| Pre-existing gastrointestinal diseases | yes | 0.809 | 0.275 | [0.269; 1.349] | 2.247 | [1.309; 3.855] | 0.011 | 0.13 |
| Body mass index | scale | 0.041 | 0.018 | [0.006; 0.075] | 1.041 | [1.006; 1.078] | 0.024 | 0.28 |

**Supplementary Table 1 (continued)**

| *Kiel-II (n=1,613)** | | | | | | | | |
| --- | --- | --- | --- | --- | --- | --- | --- | --- |
| Resilience (BRS) | scale | -0.852 | 0.131 | [-1.108; -0.596] | 0.427 | [0.330; 0.551] | <0.0001 | <0.0001 |
| No. serious or life-threatening symptoms^a^ | 1-3 | 0.342 | 0.187 | [-0.025; 0.708] | 1.407 | [0.975; 2.031] | 0.069 | 0.97 |
|  | 4-6 | 0.803 | 0.226 | [0.359; 1.247] | 2.232 | [1.432; 3.478] | 0.00043 | 0.0060 |
|  | >7 | 1.512 | 0.293 | [0.937; 2.087] | 4.535 | [2.553; 8.057] | <0.0001 | <0.0001 |
| Body weight change after infection^c^ | loss | -0.463 | 0.217 | [-0.889; -0.037] | 0.629 | [0.411; 0.964] | 0.034 | 0.47 |
|  | none | -0.683 | 0.185 | [-1.047; -0.320] | 0.505 | [0.351; 0.726] | 0.00049 | 0.0069 |
| Sex | male | -0.439 | 0.135 | [-0.704; -0.174] | 0.645 | [0.495; 0.840] | 0.0013 | 0.019 |
| Pre-existing neurologic or psychiatric disease | yes | 0.478 | 0.171 | [0.142; 0.814] | 1.612 | [1.152; 2.256] | 0.0055 | 0.077 |
| Gastrointestinal diseases | yes | 0.747 | 0.278 | [0.201; 1.292] | 2.110 | [1.223; 3.640] | 0.0086 | 0.12 |
| No. symptoms^b^ | 3-5 | -0.487 | 0.370 | [-1.212; 0.237] | 0.614 | [0.298; 1.267] | 0.19 | n.a. |
|  | 6-8 | -0.058 | 0.335 | [-0.715; 0.599] | 0.944 | [0.489; 1.821] | 0.86 | n.a. |
|  | >9 | 0.808 | 0.324 | [0.173; 1.443] | 2.244 | [1.189; 4.235] | 0.013 | 0.18 |
| Body mass index | scale | 0.028 | 0.013 | [0.003; 0.053] | 1.028 | [1.003; 1.054] | 0.031 | 0.43 |
| Pre-existing cardiovascular disease | yes | 0.356 | 0.170 | [0.024; 0.689] | 1.428 | [1.024; 1.992] | 0.038 | 0.53 |

No.: number. *Includes only participants with complete data on PCSS-R. Reference levels: ^a^no symptoms, ^b^0-2 symptoms, ^c^weight gain. ^d^P values were Bonferroni-adjusted by multiplication with the total number of predictor variables present in each sub-cohort-specific regression model. Only variables for which at least one level yielded an adjusted p value <0.05 are shown. Variables with an adjusted p value <0.05 in both sub-cohorts are highlighted in green.

**Supplementary Table 2: Significant predictors of acute severity-specific PCS-S score class in sub-cohorts Würzburg/Berlin and Kiel-II**

| **Predictor variable** | **Level** | **Regression coefficient** | | | **Odds ratio** | | **P^e^ value** | |
| --- | --- | --- | --- | --- | --- | --- | --- | --- |
|  |  | **Estimate** | **Standard error** | **95% confidence interval** | **Estimate** | **95% confidence interval** | **Unadjusted** | **Adjusted** |
| *Würzburg/Berlin (n=884)** | | | | | | | | |
| Body mass index | scale | 0.065 | 0.018 | [0.031; 0.1] | 1.068 | [1.031; 1.105] | 0.00023 | 0.0025 |
| No. serious or life-threatening symptoms^a^ | 1-3 | 0.636 | 0.234 | [0.179; 1.094] | 1.890 | [1.196; 2.987] | 0.0081 | 0.089 |
|  | 4-6 | 0.808 | 0.287 | [0.246; 1.37] | 2.243 | [1.279; 3.933] | 0.0051 | 0.056 |
|  | >7 | 1.161 | 0.338 | [0.499; 1.822] | 3.192 | [1.647; 6.186] | 0.00065 | 0.0071 |
| Pre-existing neurologic or psychiatric disease | yes | 1.047 | 0.24 | [0.576; 1.518] | 2.85 | [1.779; 4.565] | <0.0001 | <0.0001 |
| No. symptoms^b^ | 3-5 | -0.265 | 0.444 | [-1.135; 0.605] | 0.767 | [0.321; 1.832] | 0.55 | n.a. |
|  | 6-8 | 0.391 | 0.395 | [-0.384; 1.166] | 1.478 | [0.681; 3.208] | 0.33 | n.a. |
|  | >9 | 1.148 | 0.389 | [0.386; 1.911] | 3.153 | [1.471; 6.758] | 0.0045 | 0.049 |
| General anxiousness | yes | 0.418 | 0.17 | [0.085; 0.75] | 1.519 | [1.089; 2.117] | 0.014 | 0.15 |
| Age | scale | 0.013 | 0.006 | [0.002; 0.024] | 1.013 | [1.002; 1.024] | 0.018 | 0.20 |

**Supplementary Table 2 (continued)**

| *Kiel-II (n=1,613)** | | | | | | | | |
| --- | --- | --- | --- | --- | --- | --- | --- | --- |
| No. serious or life-threatening symptoms^a^ | 1-3 | 1.046 | 0.157 | [0.738; 1.355] | 2.848 | [2.092; 3.875] | <0.0001 | <0.0001 |
|  | 4-6 | 2.08 | 0.213 | [1.662; 2.497] | 8.001 | [5.27; 12.148] | <0.0001 | <0.0001 |
|  | >7 | 2.352 | 0.278 | [1.807; 2.898] | 10.508 | [6.09; 18.131] | <0.0001 | <0.0001 |
| Body mass index | scale | 0.04 | 0.014 | [0.013; 0.066] | 1.040 | [1.013; 1.069] | 0.0040 | 0.044 |
| Resilience (BRS) | scale | -0.573 | 0.105 | [-0.779; -0.368] | 0.564 | [0.459; 0.692] | <0.0001 | <0.0001 |
| Sex | male | -0.472 | 0.129 | [-0.725; -0.22] | 0.624 | [0.484; 0.803] | 0.00025 | 0.0028 |
| Education^c^ | university entrance | -0.391 | 0.14 | [-0.665; -0.118] | 0.676 | [0.514; 0.889] | 0.0058 | 0.063 |
| Pre-existing pulmonary disease | yes | 0.596 | 0.219 | [0.167; 1.026] | 1.815 | [1.181; 2.789] | 0.0098 | 0.11 |
| Pre-existing rheumatic disease | yes | 0.694 | 0.28 | [0.145; 1.242] | 2.002 | [1.156; 3.464] | 0.014 | 0.16 |
| Body weight change after infection^d^ | loss | -0.154 | 0.225 | [-0.596; 0.287] | 0.857 | [0.551; 1.333] | 0.49 | n.a. |
|  | none | -0.375 | 0.169 | [-0.706; -0.044] | 0.687 | [0.494; 0.957] | 0.027 | 0.30 |

No.: number. *Includes only participants with complete data on PCSS-S. Reference levels: ^a^no symptoms, ^b^0-2 symptoms. ^c^other school degree, ^d^weight gain. ^e^P values were Bonferroni-adjusted by multiplication with the total number of predictor variables present in each sub-cohort-specific regression model. Only variables for which at least one level yielded an adjusted p value <0.05 are shown. Variables with an adjusted p value <0.05 in both sub-cohorts are highlighted in green.
